# Interactions between the host dietary habits and the gut microbiota influence weight management

**DOI:** 10.1093/lifemedi/lnad020

**Published:** 2023-06-06

**Authors:** Yuhua Gao, Jun Lin, Frank J Gonzalez, Changtao Jiang

**Affiliations:** Department of Physiology and Pathophysiology, School of Basic Medical Sciences, Peking University, and the Key Laboratory of Molecular Cardiovascular Science (Peking University), Ministry of Education, Beijing 100191, China; Center of Basic Medical Research, Institute of Medical Innovation and Research, Third Hospital, Peking University, Beijing 100191, China; Center for Obesity and Metabolic Disease Research, School of Basic Medical Sciences, Peking University, Beijing 100191, China; Department of Physiology and Pathophysiology, School of Basic Medical Sciences, Peking University, and the Key Laboratory of Molecular Cardiovascular Science (Peking University), Ministry of Education, Beijing 100191, China; Center of Basic Medical Research, Institute of Medical Innovation and Research, Third Hospital, Peking University, Beijing 100191, China; Center for Obesity and Metabolic Disease Research, School of Basic Medical Sciences, Peking University, Beijing 100191, China; Center for Cancer Research, National Cancer Institute, National Institutes of Health, Bethesda, MD 20892, USA; Department of Physiology and Pathophysiology, School of Basic Medical Sciences, Peking University, and the Key Laboratory of Molecular Cardiovascular Science (Peking University), Ministry of Education, Beijing 100191, China; Center of Basic Medical Research, Institute of Medical Innovation and Research, Third Hospital, Peking University, Beijing 100191, China; Center for Obesity and Metabolic Disease Research, School of Basic Medical Sciences, Peking University, Beijing 100191, China

Obesity is one of the most challenging metabolic complications plaguing the world. The prevalence of obesity has increased to pandemic levels over the past 50 years [1]. Although many factors, such as genetics, could account for susceptibility to obesity, dietary habits, especially a preference for high-caloric foods, might explain most cases of obesity [1]. Dietary restriction is an increasingly popular way to lose weight, but weight regain after weight loss is a major problem, and the mechanisms behind this rebound are still largely unclear.

**Figure 1.**
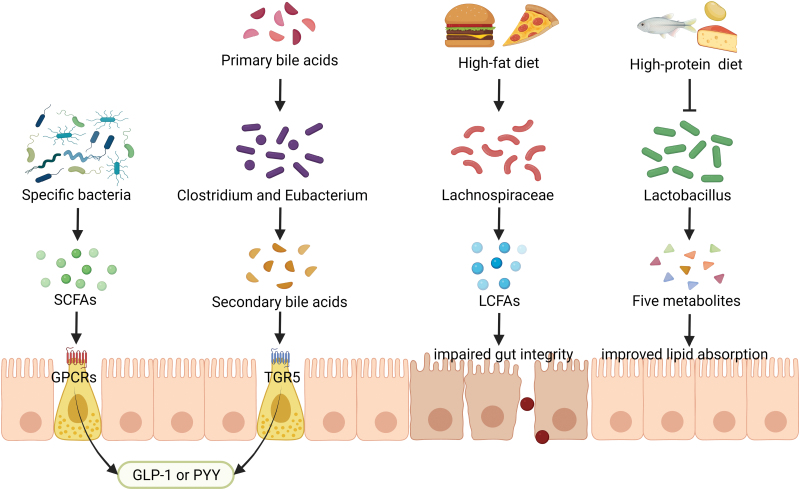
**Intestinal microbial metabolites influence host metabolism.**The human gut microbiota can produce multiple bioactive compounds that influence host metabolism. SCFAs, butyrate, propionate, and acetate, act through GPCRs expressed by enteroendocrine cells and stimulate glucagon-like peptide 1 (GLP-1) and peptide YY (PYY) release. Primary bile acids are converted to secondary bile acids by Clostridium and Eubacterium. The secondary bile acids, such as DCA and LCA, could also promote GLP-1 release to modulate host energy homeostasis and metabolism by activating TGR5. Lachnospiraceae can produce abundant LCFAs responding to HFD and exacerbate obesity through the impairment of gut integrity. Refeeding a high-protein diet after SDR prevents *Lactobacillus* growth. A joint action of five *Lactobacillus* metabolites can increase intestinal lipid absorption and fat mass.

The gut microbiota, which is frequently viewed as a “microbial organ,” is believed to be essential for the maintenance of host physiology, and the connections between the gut microbiota and human health have become clearer over the past two decades. Numerous animal studies and a handful of human studies suggest that intestinal microbial metabolites play a key role in these connections (Fig. 1). For example, short-chain fatty acids (SCFAs) affect body adiposity through various mechanisms. Notably, acetate, propionate, and butyrate induce the secretion of peptide YY (PYY) and glucagon-like peptide 1 (GLP-1) from enteroendocrine L cells through activation of the G protein-coupled receptors (GPCRs) GPR43 (FFAR2) and GPR41 (FFAR3) [2]. However, acetate was also shown to promote metabolic syndrome through a microbiota–brain–β-cell axis [3]. Additionally, secondary bile acids, such as lithocholic acid (LCA) and deoxycholic acid (DCA), which are transformed from primary bile acids by gut bacteria, can activate TGR5 and induce secretion of GLP-1 as well [4]. Several studies have revealed that Lachnospiraceae, a member of Firmicutes phylum, is highly enriched in obese individuals [5]. However, the mechanisms underlying this association have not been elucidated. Strains of the genus *Lactobacillus*, belonging to the family Lactobacillaceae, have a long history of safe and effective use as probiotics. However, whether there is a causal link between obesity and probiotics is highly controversial [6]. Lactic acid is the main end product of bacterial heterofermentative carbohydrate metabolism. l-lactate secreted by *Lactobacillus paracasei* can inhibit chylomicron secretion and promote enterocyte lipid storage [7]. Nevertheless, more research is needed to establish the relationship between lactic acid and metabolic diseases.

Recently, two publications, Takeuchi et al. and Zhong et al., reported that the interactions between host dietary habits and gut microbiota, Lachnospiraceae and Lactobacillaceae respectively, can influence weight management by producing different metabolites [8, 9].

The Takeuchi’s team previously discovered that a novel intestinal anaerobic bacterium of the family Lachnospiraceae named *Fusimonas intestini* (FI) could accelerate the development of obesity and diabetes [10]. First, they conducted a general survey of the prevalence of FI in mice and humans with metabolic disorders. FI was abundant in patients with metabolic diseases, suggesting a possible involvement in the pathogenesis. In particular, *Escherichia coli* and FI di-colonized mice showed increased body mass gain, adipose tissue weight, blood glucose levels, total serum cholesterol, and proinflammatory cytokines compared with *E. coli* monocolonized mice when fed a high-fat diet (HFD) rather than a normal chow diet. The effects were maintained in a complex microbial community, although the relative abundance was always low.

To elucidate the underlying mechanisms by which FI contributes to the pathology of obesity, the authors analyzed the metabolomic landscape in feces using gas chromatography-mass spectrometry. They investigated whether hydrophilic or lipid metabolites were altered by the combination of an HFD and FI. Through a series of data analyses, the authors found that elaidate, palmitate, and several saturated fatty acids, such as stearate and margarate, were significantly increased only when an HFD was provided together with FI. While long-chain fatty acids (LCFAs) are also microbial metabolites, few studies have investigated their relationship with metabolic diseases. The authors then quantified the production of LCFAs by FI and other representative species of human gut microbiota *in vitro*. FI and *Marvinbryantia formatexigens*, another Lachnospiraceae species, produced elaidate and consumed oleate, a cis isomer of elaidate.

Takeuchi et al. then found that dietary lipids altered the expression of FadR, a regulator of fatty acid biosynthesis, in FI to increase the production of fatty acids. By monocolonizing HFD-fed germ-free mice with FadR-overexpressing *E. coli*, the authors showed a direct role for LCFAs in metabolic diseases. After excluding the effects through direct absorption, using a series of *in vivo* and *in vitro* studies, Takeuchi et al. showed that the impairment of gut integrity led to the pathologies.

Unlike the research strategy of Takeuchi et al., Zhong et al. started with various short-term dietary restriction (SDR) models and discovered that in all 10 models, refeeding after dieting caused a rapid fat mass accumulation. Considering an unchanged energy expenditure and food intake, they found enhanced intestinal lipid absorption, increased fatty acid uptake and lipid synthesis in white adipose tissue (WAT), and decreased total lipid oxidation contributing to the fat mass accumulation. Similar to Takeuchi et al., the authors used metabolomics and found that the levels of most amino acids were significantly altered. To further investigate the potential impact of amino acids on fat mass accumulation and explore potential dietary interventions, the authors compared diets with varying protein levels after SDR and demonstrated that a high-protein diet could attenuate intestinal lipid absorption. By analyzing the microbiota composition by 16S rRNA gene sequencing of feces, the authors identified an enrichment of the genus *Lactobacillus* that contributed to enhanced lipid absorption. The joint effect of five metabolites, DL-3-phenyllactic acid (PLA), 4-hydroxyphenyllactic acid (HPLA), 2-hydroxyisocaproic acid (HICA), 2-hydroxy-3-methylbutyric acid (HMBA), and indole-lactic acid could explain the impact on obesity.

Surprisingly, Takeuchi et al. reported that FI, a Lachnospiraceae species, exacerbated HFD-induced obesity via LCFAs through the impairment of gut integrity. However, Zhong et al. found that the genus *Lactobacillus* enhanced intestinal lipid absorption and fatty acid uptake in WAT and lead to weight regain after dieting by producing five metabolites. By focusing on different animal models, microbiota and metabolites, the two groups both show that the interactions between host dietary habits and gut microbiota play an important role in weight management.

One relevant point for discussion centers on the mechanisms by which the metabolites are increased and their modes of action. Previous work in this area has primarily focused on the roles of SCFAs and other metabolites in metabolic disorders, with few studies examining LCFAs, especially trans-unsaturated fatty acids, and lactic acid. Moreover, most studies have concentrated on the action of an individual metabolite, while Zhong et al. demonstrated the importance of a combination of five metabolites, all of which were necessary. However, one limitation of both papers is the lack of genetic studies. Importantly, the absence of gene manipulation on Lachnospiraceae failed to demonstrate the importance of FI-fadR. The reason why dietary fat only influenced the expression of FI-fadR rather than *E. coli*-fadR remains to be determined. In addition, it is unclear whether there is a gene(s) responsible for the production of the five metabolites by *Lactobacillus*, and thus further analysis is needed. Studies on genetic modification may provide insights for future treatments. It would also be of interest to examine the production of the fatty acids and lactic acid and their physiological or pathological effects in a broader range of microorganisms.

These two studies inspire several new questions as to the precise mechanisms underlying the functions of the microbiota and metabolites in obesity and metabolic diseases. Although the family Lachnospiraceae is associated with obesity and insulin resistance in several studies, Takeuchi et al. are the first to provide a mechanism related to the intestinal barrier. It is possible that other mechanisms are also involved, especially in a complex microbial community, and a precise molecular mechanism may also be required. The way in which five metabolites found by Zhong et al. affect lipid absorption is even less clear.

In conclusion, these two studies show that the interactions between host dietary habits and gut microbiota play an important role in diet-induced obesity and weight regain after dietary restriction. The novel mechanisms and more in-depth knowledge will provide insights to develop new therapies for treating obesity.
